# Who Opts Out? The Customisation of Marriage in the German Matrimonial Property Regime

**DOI:** 10.1007/s10680-022-09613-8

**Published:** 2022-03-23

**Authors:** Theresa Nutz, Anika Nelles, Philipp M. Lersch

**Affiliations:** 1 GESIS – Leibniz Institute for the Social Sciences, B6, 4-5, 68159 Mannheim, Germany; 2 Department of Social Sciences, Humboldt-Universität zu Berlin, Unter den Linden 6, 10099 Berlin, Germany; 3 DIW Berlin, Mohrenstr. 58, 10117 Berlin, Germany

**Keywords:** Matrimonial property regimes, Marriage cohorts, Marital contracts, Economics of marriage, Intra-couple gender inequality, Prenuptial agreements

## Abstract

This study examines the prevalence of marital contracts across marriage cohorts (1990–2019) in Germany. We further investigate the characteristics of spouses who signed a marital contract. Using cross-sectional data from the German Family Panel (pairfam, 2018/19), we employ complementary log–log and multinomial logistic regression models to predict the prevalence and the type of marital contracts. The results show that 5% of all married couples opt out of the default matrimonial property regime by signing a contract in Germany. Differentiating between contract types, most couples either specify a separation of property (40%) or modify the default community of accrued gains (31%). We find an increase in the prevalence of marital contracts across marriage cohorts. The decision to opt out of the default is strongly positively associated with self-employment that often requires the customisation of asset ownership structures within marriage. Married individuals with prior divorce experiences are more likely to opt for the separation of property, indicating that the awareness of the economic consequences of divorce promotes the individualisation of marriage. Our results are in line with the cross-national trend towards customised marriages, although the trend in Germany is less pronounced than in other countries.

**Supplementary Information:**

The online version contains supplementary material available at 10.1007/s10680-022-09613-8.

## Introduction

Despite the increase in alternative living arrangements in the last decades, marriage remains a central institution in modern societies. Regarding social policy, marriage constitutes a crucial legal status in many countries that offers financial privileges and a security function (Hamilton, 2004). How marriage legally ties two partners and the state together in the economic domain is codified in matrimonial property regimes. During marriage, the property regime determines spouses’ ownership of and access to economic resources. At divorce, the property regime regulates the division of economic resources and, hence, shapes individuals’ future economic situation—including their need for welfare support. As women commonly experience higher economic vulnerability than men, the property regime is of particular importance for women’s economic positions within the marital family and potentially perpetuates their economic disadvantage both during and after marriage.

In most countries, couples can either choose the default matrimonial property regime or opt out with a legally binding marital contract. Marital contracts can include various legal agreements that address the relations during marriage and—often more importantly—the financial consequences of divorce (Dutta, 2012; Smith, 2003). Whereas marital contracts might be one way of expressing economic autonomy within the couple, they might also reinforce existing economic inequalities by further impairing the financial situation of the more vulnerable spouse, most often the wife (Thompson, 2018). Despite their far-reaching consequences, the predictors as well as the prevalence of marital contracts remain unclear. For most countries, numbers on marital contracts rely on crude estimates or outdated data (Rainer, 2007). Also in Germany, our country case, couples signing marital contracts are not systematically registered (Mahar, 2003).

Building on a wide rational choice perspective, sociological literature on the individualisation of marriage, and empirical evidence regarding changes in matrimonial property regimes from other European countries (e.g. Fraboni & Vitali, 2019; Frémeaux & Leturcq, 2018), our research interest is in understanding the choice of matrimonial property regimes in Germany. We follow a within-couple perspective to address three research questions: (1) How prevalent are marital contracts in Germany? (2) How did the prevalence of marital contracts change across marriage cohorts between 1990 and 2019? (3) Which couples sign which types of marital contracts? To answer these questions, we draw on cross-sectional data from pairfam—The German Family Panel, in which we implemented an interview module on marital contracts in 2019. In the first analytical step, we exploratively map the prevalence of marital contracts. We additionally compare the estimates with data from the 2016 wave of the Socio-Economic Panel Study Innovation Sample (SOEP-IS) and the 1988 and 2019 waves of the SOEP. In a second step, we test specific expectations about cohort change and predictors of marital contracts, such as socioeconomic differences at the couple level or prior divorce experiences, using complementary log–log and multinomial logistic regression models.

Our contributions to the literature on marital contracts are twofold. First, we draw on unusually rich survey data from pairfam. The data combine detailed information on the socio-demographic background with comprehensively measured variables on relationships and the family background of both partners. Compared with other recent German survey^1^ and international register data (e.g. for Italy, see Fraboni & Vitali, 2019), pairfam provides unique data to examine the predictors of signing a marital contract, further allowing to differentiate between contract types. The differentiation helps us to better understand the two distinct functions of marital contracts, which might either promote spouses’ economic independence or aim at integrating economic resources—both with crucial consequences for the economic well-being of women and men during and after marriage.

Our second contribution adds to the empirical literature on marital contracts by introducing the German case with a unique matrimonial property regime paired with conservative family policies. In Germany, the crude divorce rate, defined as the annual number of divorces per 1,000 population, decreased over the past 10 years and corresponded to the EU average of 1.8 in 2019 (Eurostat, 2021). In contrast, the crude marriage rate increased to 5.0 in 2019 and exceeded the EU average of 4.3. Germany’s default property regime, the *community of accrued gains,* combines the separation of property during marriage, where spouses’ personal wealth and gains remain in individual ownership, with elements of the community of property after marriage. In the event of divorce, the surplus gains accrued during marriage are split equally. Germany therefore differs from most European countries, which have a *community of acquisitions* regime, where all newly acquired assets during marriage are joint property (further described below).^2^ As women are commonly the economically weaker spouses due to the gender-unequal division of labour during marriage, they experience a comparably high economic vulnerability under the German property regime. That is because spouses have no legal rights to participate in the other’s wealth by default in Germany (Rotino, 2015), which is inconsistent with its gender-traditional welfare system, where the spouse focusing on unpaid work should be economically protected through marriage.

Countries differ strongly in the financial and emotional costs of opting out of the default property regime through a marital contract. Regarding financial costs, marital contracts in Germany are only legally binding if they are certified by a notary, which comes with additional legal fees. In contrast, Italy allows choosing between property regimes at marriage at no additional costs (Bayot & Voena, 2014). Regarding emotional costs caused by marital contracts perceived as a potential signal of mistrust (Smith, 2003), many countries, such as Spain (Brassiolo, 2013), the Netherlands, and Scandinavian countries (Smith, 2003), have experienced increasing customisation of marriage through the general acceptance of alternative legal partnership arrangements. This development likely decreases the emotional costs of opting out of the default matrimonial property regime. For instance, France has experienced a transition to a ‘customised marriage’ through the *pacte civil de solidarité* (PACS) as a form of registered cohabitation (Frémeaux & Leturcq, 2018). This stands in contrast to Germany, where the default community of accrued gains exists since 1957 without other legally recognised partnership forms for different-sex couples.

## Background

### Types of Matrimonial Property Regimes and Marital Contracts

The legal regulation of marriage is an important aspect of family policy that is crucial for the economic consequences of marriage and divorce. In addition to the division of wealth, most countries regulate the adjustment of pension rights, post-marital maintenance, and alimony (Dutta, 2012; Radenacker et al., 2019). The regimes either follow the community or the separation of property, with modifications being common. Most European countries follow the community of acquisitions (Rotino, 2015). Under this regime, individuals’ wealth accrued before marriage as well as inheritances received during marriage remain in personal ownership, whereas other assets acquired during marriage are spouses’ joint property. At divorce, the jointly accumulated assets are split equally among both ex-spouses. See Table 1 for an overview of the default matrimonial property regimes in Europe.

**Table 1:** Matrimonial property regimes in Europe

Community of acquisitions			Community of accrued gains			Separation of property			Community of property	
*Spouse 1*		*Spouse 2*	*Spouse 1*		*Spouse 2*	*Spouse 1*		*Spouse 2*		*Couple*
EUR 10,000	Wealth at marriage entry	EUR 40,000	EUR 10,000	Wealth at marriage entry	EUR 40,000	EUR 10,000	Wealth at marriage entry	EUR 40,000	Wealth at marriage entry	EUR 50,000
			**↓**		**↓**	**↓**		**↓**		**↓**
EUR 10,000 (individual) + EUR 130,000 (joint)	Wealth at divorce	EUR 40,000 (individual) + EUR 130,000 (joint)	EUR 60,000	Wealth at divorce	EUR 120,000	EUR 60,000	Wealth at divorce	EUR 120,000	Wealth at divorce	EUR 180,000
EUR 65,000	Personal gains	EUR 65,000	EUR 50,000	Personal gains	EUR 80,000	EUR 50,000	Personal gains	EUR 80,000	Personal gains	EUR 130,000
EUR 75,000 = EUR 10,000 + EUR 65,000	Wealth after divorce	EUR 105,000 = EUR 40,000 + EUR 65,000	EUR 75,000 = EUR 60,000 + ½ (EUR 80,000–EUR 50,000)	Wealth after divorce	EUR 105,000 = EUR 120,000–½ (EUR 80,000 –EUR 50,000)	EUR 60,000	Wealth after divorce	EUR 120,000	Wealth after divorce (individual)	EUR 90,000 = ½ (EUR 180,000)
Default in Belgium, Slovenia, France, Luxemburg, Spain (with regional variation), Estonia, Latvia, Malta, Czech Republic, Poland, Hungary, Italy^b^, Lithuania, Croatia, Bulgaria, Portugal, Slovakia, Romania (Rotino, 2015)			Default in Germany, Cyprus, Greece (Rotino, 2015)^a^ Similar calculation under the *deferred community of property* in Sweden, Finland, Denmark, and Austria (Rotino, 2015)			Default in Catalonia, Balearic Islands (Brassiolo, 2013; Rotino, 2015)			Default in the Netherlands (Rotino, 2015)	

^a^In contrast to Germany, gains are not divided in half in Greece and Cyprus but proportionally to individuals’ contributions made during marriage

^b^Although Italian couples can choose between the community of acquisitions and the separation of property at marriage, the statutory default matrimonial property regime is the community of acquisitions

In Germany, spouses marry per default under the community of accrued gains (*Zugewinngemeinschaft;* §1363 of the German Civil Code). Individuals’ wealth (including inheritances and transfers) accrued before and during marriage remains in personal ownership during marriage. In the event of divorce, the surplus gains^3^ accrued during marriage are divided equally between both ex-spouses. The regime should ensure that both spouses benefit equally from the wealth acquired during marriage. Unlike the community of acquisitions, however, the economically weaker spouse is not equally benefitting from the partner’s wealth during marriage. This prevents them from managing assets and building personal wealth (Nake, 2013; Rotino, 2015), which may reduce bargaining power and well-being. Prior literature on matrimonial property regimes highlights that both the community of accrued gains and the community of property aim at regulating the divorce of marriages with a (most often male) single earner, little wealth, and children (Fraboni & Vitali, 2019; Langenfeld & Milzer, 2019). These regimes intend to protect the economically weaker spouse by considering most assets as joint. For female-breadwinner couples, the separation of property might be considered more beneficial because wives’ need for economic protection from the husband is reduced (Fraboni & Vitali, 2019). For other couple types, however, the economic benefits of a modification regulating the division of jointly accrued gains in the case of divorce might be highest.

To opt out of the default, couples can agree upon a marital contract either before (prenuptial) or during marriage (postnuptial) (Dutta, 2012). In Germany, couples commonly opt for the separation or the community of property. Under the separation of property, both spouses remain the sole owners of their personal wealth during and after marriage. In the event of divorce, no compensation takes place. Under the community of property, both spouses’ assets are merged into one pool of equally held property. With divorce, the commonly held property is divided equally. Marital contracts can also flexibly modify parts of the community of accrued gains. For instance, they can regulate alimony payments, exclude certain property and pension rights from the compensation, or define the value of assets at the beginning of marriage to avoid uncertainty in the calculation of compensation claims in case of divorce (Dutta, 2012).

### Prevalence of Marital Contracts and Trends Across Countries

Due to country differences in the legal regulation of marriage and the existence of marriage registers, estimates on the prevalence of marital contracts are only partially comparable across countries. Similar to many other countries, marital contracts are not officially registered in Germany (Mahar, 2003). Consequently, there is a lack of reliable administrative data, as the register only records marital contracts on a voluntary basis (Dutta, 2012). Estimates from these registers from the 1980s suggest that less than 10% of married couples in Germany have a marital contract (Schreiber, 1983; Stach, 1988). Whereas more recent survey data estimate the share of couples signing a marital contract at 7%, they also show that 18% of married individuals believe that they do not live under the default regime (Wippermann, 2013). These inconsistencies suggest that misconceptions about the meaning and implications of matrimonial property regimes are widespread.

In France, roughly 18% of newlywed couples have signed a prenuptial agreement in 2010 (Frémeaux & Leturcq, 2018). At 25%, prenuptial agreements are also common in the Netherlands, the only European country with a default community of property regime (Page (2001), as cited in Smith (2003)). For the United States, the estimated share of couples with marital contracts varies between 5 to 10% (Mahar, 2003; Marston, 1997), with differences in the matrimonial property regimes between states. Italy is a special case with comprehensive marriage register data, including the entire population of marriages since 1995 (Fraboni & Vitali, 2019). The share of newlywed couples opting for the separation of property was around 67% in 2011 (Bayot & Voena, 2014). The high prevalence of couples choosing the separation of property can be partly explained by the regulation that couples at marriage are asked to decide between two matrimonial property regimes without signing a costly prenuptial contract (Fraboni & Vitali, 2019; Ruiu & Breschi, 2017).

In addition to between-country differences, studies also identified general trends over time. In Italy, Fraboni and Vitali (2019) find a strong decrease in couples choosing the community of property regime over marriage cohorts. Whereas 59% of couples married in 1995 opted for the community of property regime, 29% of newlyweds chose this regime in 2015. Frémeaux and Leturcq (2018) report a rise in the incidence of the separation property regime in France since the 1970s. This increase corresponds to the overall rates of signed marital agreements in the same period. Building on the individualisation of marriage, we will theoretically explain these trends in the following section.

### Theoretical Framework

From a wide rational choice perspective, individuals enter marriage if the expected utility exceeds the costs associated with marrying the current partner (Becker, 1973). The cost–benefit considerations of marriage are influenced by economic and non-economic factors, such as legal protection for joint financial investments, expected intimacy, or financial independence (Cherlin, 2004). Linked with the decision to marry is the decision to sign a marital contract, which alters the benefits and costs of marriage. However, the marital contract itself comes with costs—legal fees and emotional costs of disagreements over the contract’s content and of the contract as a signal of mistrust (Smith, 2003). Benefits and costs may be unequally distributed within couples, particularly spouses with large resource differentials may have contrasting preferences. In addition, the benefits and costs of marital contracts vary over time, as preferences and legal regulations differ.

Most couples marry without explicitly considering opting out of the default regime. This may be because of fundamental trust in the institution of marriage and its protective function (Wippermann, 2013). Furthermore, legal regulations are subject to misconception (Rowlingson & Joseph, 2010). For instance, there is a common misunderstanding about the implications of the German default regime. The community of accrued gains is often confused with the community of property, claiming that both spouses own assets equally during marriage (Rotino, 2015). Furthermore, many spouses are not adequately informed about their matrimonial property regime and respective regulations (Wippermann, 2013). Such subjective beliefs and misinformation are likely to influence individuals’ choices of marital contracts.

#### Individualisation of Marriage

Examining the predictors of marital contracts with a cost–benefit calculus must be seen embedded in historical changes in the meaning of marriage, divorce law, and female employment. Historically, marital contracts have been an opportunity to protect wives from their husbands’ financial power (Smith, 2003). Since the introduction of the no-fault divorce in the 20th century in many western countries and because of women’s growing financial independence due to increased labour force participation; however, marital contracts can be seen as a protection for both spouses. Also, the meaning of marriage shifted from institutional in the 19th over companionate during the 20th century to individualised marriage thereafter, which emphasises spouses’ personal choice and self-development (Cherlin, 2004).

The implications of the individualisation of marriage are twofold (Yodanis & Lauer, 2014). First, during the ‘Second Demographic Transition’ (van de Kaa, 1987), constraints caused by strict ideas, norms, and informal rules related to marriage have been weakened. Thus, spouses’ behaviour is increasingly determined by their own interests instead of the institution of marriage. Second, spouses put more emphasis on their individual preferences instead of acting as an interdependent couple unit.

Marital contracts may increase the perceived benefits of marriage by providing a legal and institutionalised opportunity for its customisation. The individualisation of marriage is linked to the preference of keeping money separate and a reduced need for economic support. Emotional companionship and personal autonomy gain in importance. Further, spouses in individualised marriages may anticipate marital breakup. Therefore, they may minimise the economic risks associated with marriage by governing the financial outcomes of divorce in advance.

Because marital contracts can be flexibly designed, they allow couples to make specific arrangements instead of being determined by one default option. This tendency towards customisation of marital contracts is embedded in profound changes in the role of divorce law (Cherlin, 2004). The introduction of the no-fault divorce and reforms of alimony payments in many western countries mark a general trend towards the ‘privatisation’ of marriage, which also favours marital contracting (Smith, 2003).

#### Economic Resources, Division of Labour, and Marital Contracts

With increasingly individualised marriages, the cost–benefit considerations of opting out of the default may be driven by a variety of economic and non-economic factors. Individuals should prefer the matrimonial property regime providing the highest individual expected utility. Generally, the benefits of the separation of property should increase with individuals’ expected financial resources. The spouse with higher expected resources may prefer not to share them during marriage and, particularly, at divorce (Wippermann, 2013). In addition, one spouse’s resources relative to the other may affect their weight in decisions on the arrangements to be included in the marital contract (Blood & Wolfe, 1960).

Resource differentials should be particularly large if partners had unequal opportunities to establish themselves in the labour market and face unequal potentials to accumulate wealth in the future. Of crucial importance are age differentials. Differently aged spouses are likely to have accumulated distinct levels of assets and income levels at marriage entry, as older spouses have been employed for a longer time (Smith, 2003). Furthermore, educational differences set the lower-educated spouse at economic disadvantage (Fraboni & Vitali, 2019). Education also entails non-economic resources, such as negotiating skills, that may be used to convince the spouse of one’s preferences regarding marital contracting. We therefore expect couples with differentials in age or education to be more likely to opt for the separation of property than couples of similar age or education. Under a gender-traditional division of labour, however, the tendency to agree on a separation of property might be outweighed by a preference for economic sharing in these couples, as we discuss in the following.

On the one hand, for couples with a traditional division of labour, the potential to accumulate wealth is often higher for men than for women (Deere & Doss, 2006). Women are likely to be the economically weaker spouses in these couples and might strongly depend on their husbands’ resources. In this situation, we expect men to have a larger interest in opting for the separation of property to protect their assets both throughout and beyond marriage. One the other hand, the separation of property would undermine their role as breadwinners contributing to the whole family’s economic well-being, with compensation for the unpaid work of their wives. Following this reasoning, male-breadwinner couples may be more likely to make couple-specific investments and should, therefore, either opt for the community of accrued gains or the community of property. In the specific German case, the welfare state with joint taxation and tax reductions for married couples with unequal earnings incentivises couples to form one economic unit (Dingeldey, 2001), making it more likely that male-breadwinner couples opt for the default community of accrued gains.

In addition to spouses with large resource differentials, the separation of property may be also preferable for dual-earner couples (Bayot & Voena, 2014). With two full-time employed earners, both partners tend to be economically independent and more inclined to individualised marriage. Despite often having relatively similar levels of financial resources, dual-earner couples might prefer the separation of property, as there is less need for economic sharing and the financial compensation of one spouse focusing on unpaid work. Prior literature finds the separation of property to be associated with decreasing couple-specific investments and a higher female labour market participation (Brassiolo, 2013; Frémeaux & Leturcq, 2018). We therefore expect dual-earner couples to be likely to opt for the separation of property.

Furthermore, self-employed individuals may aim to avoid economic conflicts by keeping wealth separately (Fraboni & Vitali, 2019; Ruiu & Breschi, 2017). On the one hand, self-employed individuals may protect business assets from their spouses through a marital contract (Frémeaux & Leturcq, 2018). Otherwise, divorce could lead to the disposal of professional or business assets due to legal compensation claims. On the other hand, the marital contract protects the property of the non-self-employed spouse because it cannot be seized as collateral in case of bankruptcy, as it is the case under the community of property. Thus, couples with a self-employed spouse should be more likely to opt for the separation of property than couples without self-employed spouses.

#### Prior Relationship Experience and Family Background

Spouses’ partnership biographies may influence the perceived costs and benefits associated with marital contracts. First, experiences of divorce or cohabitation dissolution may raise awareness for a relationship breakdown and, therefore, increase the benefits of marital contracts to prepare for such contingencies. Second, having experienced negative economic consequences of a previous divorce or dissolution may increase the perceived utility of protecting one’s wealth through marital contracts in the future. Third, remarried spouses with children from former relationships are found to prefer keeping wealth separately to protect their biological children’s prospective inheritances (Burgoyne & Morison, 1997). Fourth, Wippermann (2013) shows that a considerable share of women and men wrongly assume that they do not have to fulfil any economic responsibilities towards their former spouse after divorce. Having experienced prior divorce may therefore increase knowledge of the legal situation and the economic consequences. Correspondingly, spouses in higher-order marriages are found to be better informed about the meaning of the statutory matrimonial property regime (Federal Ministry for Family Affairs, Senior Citizens, Women & Youth of Germany, 2014).

Next to the own experience of partnership breakdown, parental divorce or separation may contribute to the choice of marital contracts for similar reasons. It may expand knowledge of economic risks and legal regulations of divorce, thereby increasing the perceived benefits of marital contracts. Billari and Liefbroer (2016) find the experience of parental separation or divorce being associated with a slower transition to marriage due to more negative feelings compared with children of stable-married parents. Overall, we expect own or parental previous divorce or dissolution experiences to increase the likelihood of the separation of property.

Also the marriage ritual might predict the choice of matrimonial property regime. Whereas a religious component is mostly unnecessary for a valid civil marriage, many marriage ceremonies combine both aspects (Hamilton 2004). Couples choosing a religious ritual might comply with a traditional understanding of life-long marital commitment. Therefore, we expect couples choosing a religious marriage to be more likely to opt for the community of property. However, prior research from Italy finds no clear association between the marriage ritual and the property regime, suggesting that wedding ceremonies reflect symbolic traditions rather than religious beliefs (Fraboni & Vitali, 2019; Ruiu & Breschi, 2017).

## Data, Measures, and Method

### Data

Analyses are based on data from wave 11 of the German Family Panel surveyed in 2018/19 (pairfam) (Brüderl et al., 2020). The study started in 2008/2009 with a sample of anchor persons who have been interviewed annually.^4^ With its multi-actor-design, pairfam additionally surveys the family members (partners, children, parents) of the anchor person. The data include information on anchor persons from three birth cohorts (1971–1973, 1981–1983, 1991–1993). In wave 11, we implemented an interview module on marriage contracts. Besides the choice of matrimonial property regime, pairfam data collected comprehensive information on both spouses’ marriage characteristics, prior family experiences, division of labour, parents’ characteristics, and demographics.

There are three other publicly available surveys with information on marital contracts for Germany, which we draw on for descriptive analyses as a benchmark for the pairfam data. We additionally use data from the 2016 wave of the Socio-Economic Panel Study Innovation Sample (SOEP-IS, Richter & Schupp, 2015) and the waves 1988 and 2019 of the SOEP (version 36, Goebel et al., 2019). Given our focus on marital contracts, we restrict the analytical sample to currently married couples aged 18 years or older. Due to different legal historical regulations, we exclude same-sex marriages and couples who married abroad from the analyses. The analytical sample includes 2,880 couples.

### Measures

Our binary dependent variable is the presence of a marital contract in contrast with remaining in the default matrimonial property regime. In additional specifications, we differentiate between three different contract types (separation of property, modified community of accrued gains, and community of property or other contract). The choice of the independent variables follows the theoretical discussion in the previous sections. With the marriage cohorts (1990–1999, 2000–2009, 2010–2019), we test if the prevalence of marital contracts has changed over time. To measure educational differences between spouses, we distinguish between couples with both spouses having either the same (reference group) or distinct levels of education (higher education of man or woman). We capture age differentials between both spouses in three categories, differentiating between couples in which either both spouses are of similar age (± 3 years; reference group), couples in which the man (> 3 years) and those in which the woman is older (> 3 years).^5^ To measure the current division of labour within couples, we differentiate between dual-earner (reference group), male and female-breadwinner, and jobless couples. We also include a measure of at least one partner currently being self-employed.

We control for the anchor’s birth cohort (1971–1973 (reference group), 1981–1983, 1991–1993) and the region the couple currently lives in (Eastern or Western Germany) because of a historically different default matrimonial property regime, the community of acquisitions, in the former German Democratic Republic until 1990. We also include the length in premarital cohabitation since cohabiting partners are more likely to manage their economic resources separately, which they might continue during marriage (Vogler et al., 2006).

### Method

To examine the predictors of marital contracts, our analytical approach follows two steps. First, we fit a regression model with a binary dependent variable indicating the prevalence of a marital contract with a complementary log–log (cloglog) function. We estimate nested models and successively include our independent variables according to the time of measurement in order to consider the possibility of reverse causality. Due to the dyadic nature of marital contracts, we run the analyses at the couple level. The cloglog model is used as an alternative prediction model over the conventional logit and probit models for rare events data. Accounting for missing data, only 5.0% of the couples in the underlying analytical sample have signed a marital contract. Unlike logit and probit functions, the cloglog function is not symmetric but skewed to the right. Hence, it considers the rarity of marital contracts and does not underestimate its probability (King & Zeng, 2001).^6^

Second, we estimate multinomial logistic regression models to examine the predictors of the type of marital contract. We predict the occurrence of marital contracts for couples having agreed on the separation of property, a modified community of accrued gains, and the community of property (together with other contracts) compared with the reference group of couples having no contract. For the analyses, we use the statistical software R (version 4.0.2, R Core Team, 2020) and apply multiple imputation using the Amelia II procedure (Honaker et al., 2011). A total of *M* = 30 imputations were created (Rubin, 1976; see Table A2 in the online appendix for an overview of missing values).

**Table 2:** Descriptive statistics of all analytical variables

	Marital contract			No marital contract			Group difference^a^
	Mean/Prop	SD	N	Mean/Prop	SD	N	
*Contract type:*							
Community of property	.18	.39	144				
Modified comm. of accrued gains	.31	.46	144				
Other contract	.12	.32	144				
Separation of property	.40	.49	144				
*Time of contract (ref. postnuptial)*							
Prenuptial agreement	.63	.48	138				
*Marriage cohort:*							
1990–1999	.06	.23	144	.11	.31	2,734	− 0.06***
2000–2009	.43	.50	144	.34	.47	2,734	0.11*
2010–2019	.51	.50	144	.55	.50	2,734	− 0.05
*Age difference:*							
Same age	.53	.50	143	.59	.49	2,706	− 0.06
Man older	.42	.50	143	.36	.48	2,706	0.06
Woman older	.05	.22	143	.05	.21	2,706	0.00
*Educational difference:*							
Same education	.59	.49	143	.59	.49	2,723	0.03
Man higher	.15	.36	143	.18	.39	2,723	− 0.04
Woman higher	.25	.44	143	.22	.42	2,723	0.01
*Divorced (ref. first marriage):*							
Higher-order marriage	.15	.36	142	.12	.33	2,659	0.10*
*Parental divorce (ref. no divorce):*							
Parental divorce	.23	.42	88	.21	.41	1,727	0.06
*Dissolution (ref. no dissolution):*							
Cohabitation dissolution	.22	.41	144	.24	.43	2,660	0.04
*Type of marriage (ref. civil):*							
Civil and church marriage	.43	.50	92	.35	.48	1,716	0.01
*Division of labour:*							
Dual-earner	.33	.47	144	.28	.45	2,725	0.07
Male breadwinner	.56	.50	144	.64	.48	2,725	0.01
Female breadwinner	.06	.23	144	.03	.17	2,725	0.01
Jobless	.06	.23	144	.05	.22	2,725	− 0.09
*Self-employed (ref. not self-empl.):*							
Self-employed	.38	.49	144	.12	.33	2,727	0.25***
*Region (ref. Western Germany):*							
Eastern Germany	.11	.32	144	.25	.43	2,735	− 0.09***
Years in premarital cohabitation	2.56	2.57	143	3.17	3.30	2,714	− 0.48*

^a^Group differences weighted by population weights

*p < .05, **p < .01, ***p < .001 for two-tailed tests of group differences in proportion or means

Due to the cross-sectional nature of our data, we face the problem of selective “mortality” of marriages with marital contracts. We likely underestimate the share of couples opting out of the default because marriages with marital contracts might separate faster (Rainer, 2007). We therefore conducted additional analyses based on SOEP-IS data that show only modest differences in the prevalence of marital contracts among currently married respondents and divorced or separated respondents. Among the subsample of married respondents, 6.2% reported to have a marital contract, which is similar to the prevalence of 5.0% of marital contracts in our analytical sample from pairfam. Whereas respondents in the pairfam study were not asked about marital contracts in previous marriages, 9.5% of the currently divorced or separated respondents in the SOEP-IS reported they had a marital contract in their last marriage (see Figure A1 in the online appendix). Thus, while our analyses are likely to slightly underestimate the share of couples with a marital contract at the time of marriage, we assume that our main conclusions are not affected by this selection.

## Results

### Descriptive Results

In the following, we descriptively compare married couples with and without marital contracts (see Table 2). Among couples with a marital contract (5% of the underlying sample), most choose the separation of property (40%) or a modified community of accrued gains (31%). Around 18% of these couples opt for a community of property and additional 12% choose customised regimes not corresponding to the three regime types. Around 63% of all contracts are prenuptial contracts, that is, agreements concluded before marriage.

Concerning the distribution across marriage cohorts, we find that around half of all couples in our sample with and without marital contracts married in the 2010s. Whereas the proportion of the oldest marriage cohort (1990–1999) is significantly smaller among couples with marital contracts compared with couples without a contract, the share of couples married in the 2000s is significantly larger among those with marital contracts. We find no statistically significant differences for couples married in the 2010s. Regarding age and educational differences, we do not find statistically significant group differences either. Most couples have the same educational level (59% in both groups) and are of same age (53% with contract, 59% without contract).

Whereas the experience of a divorce occurs significantly more frequently among couples with a contract (15% with contract, 12% without contract), we do not find statistically significant group differences for parental divorce. Also, the proportion of couples having both a civil and a church wedding ceremony is not significantly different for couples with and without a marital contract (43% with contracts, 35% without contracts). We further do not find statistically significant group differences in the type of division of labour, with male breadwinner couples being the most frequent employment constellation in both groups (56% with contracts, 64% without contracts). At 38%, self-employment of at least one spouse is significantly more likely in couples with than without a marital contract (12%). Regarding regional differences, we find the proportion of couples from Eastern Germany to be significantly smaller for those with than without a contract (11% with contracts, 25% without contracts).

**Fig. 1:**
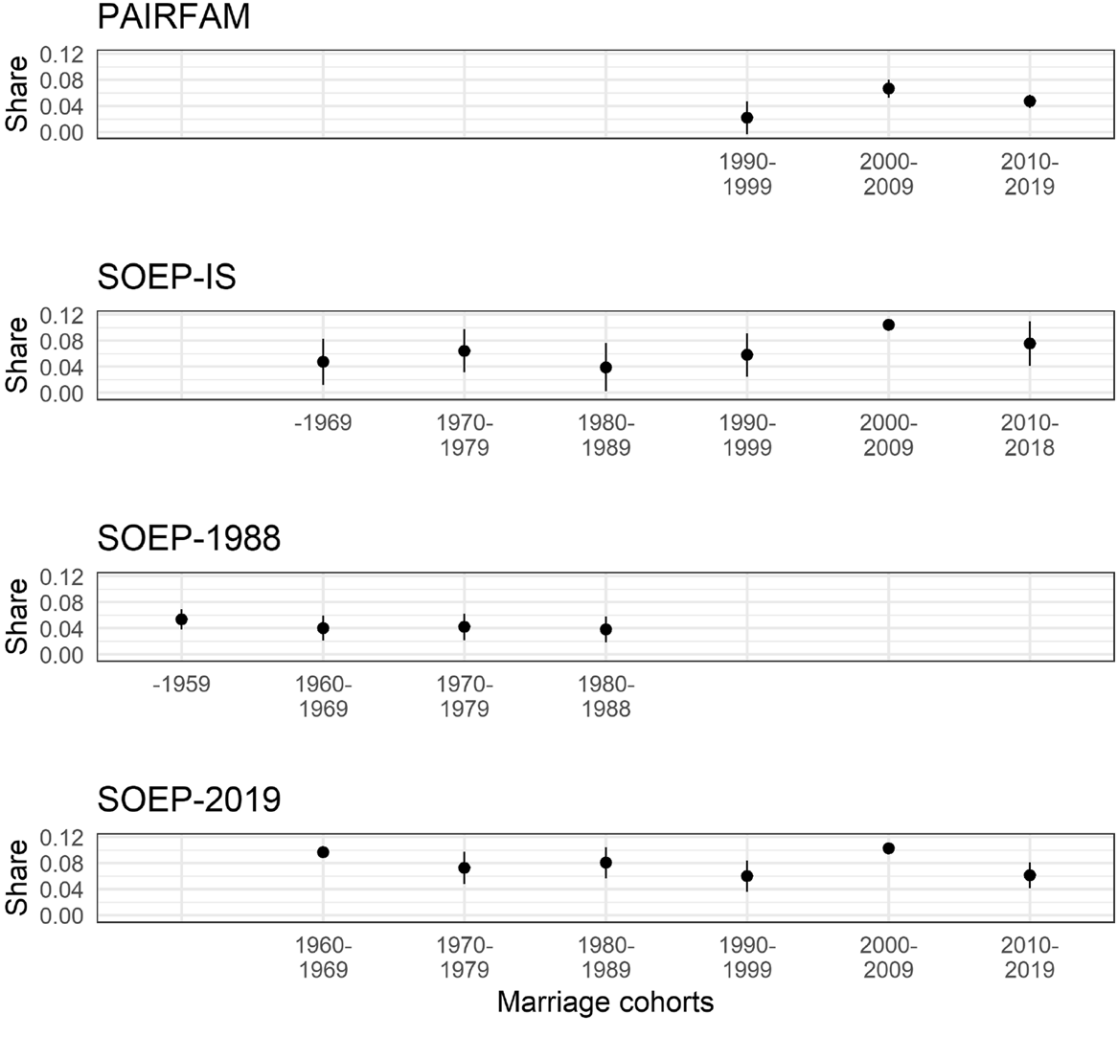
Share of couples with marital contract by marriage cohort *Note*: Lines indicate 95% confidence intervals

Figure 1 presents the shares of couples with marital contracts of all married couples by marriage cohort. The upper panel shows estimates based on pairfam data, whereas the middle and lower panels present comparative estimates from SOEP-IS, SOEP-1988, and SOEP-2019. The average share of marital contracts in Germany ranges from 5 to 10% over time. Before 1990, the shares also vary between 5% (see SOEP-1988 and SOEP-IS) and 10% (SOEP-2019). We observe an increase in the shares of marital contracts between the 1990s and 2000s marriage cohorts. In the 2000s, the estimated share of marital contracts lies around 8 and 10% (see pairfam, SOEP-IS, and SOEP-2019). The 2010s marriage cohorts are less likely to have a marital contract than the 2000s cohorts.

### Multivariable Results

**Fig. 2:**
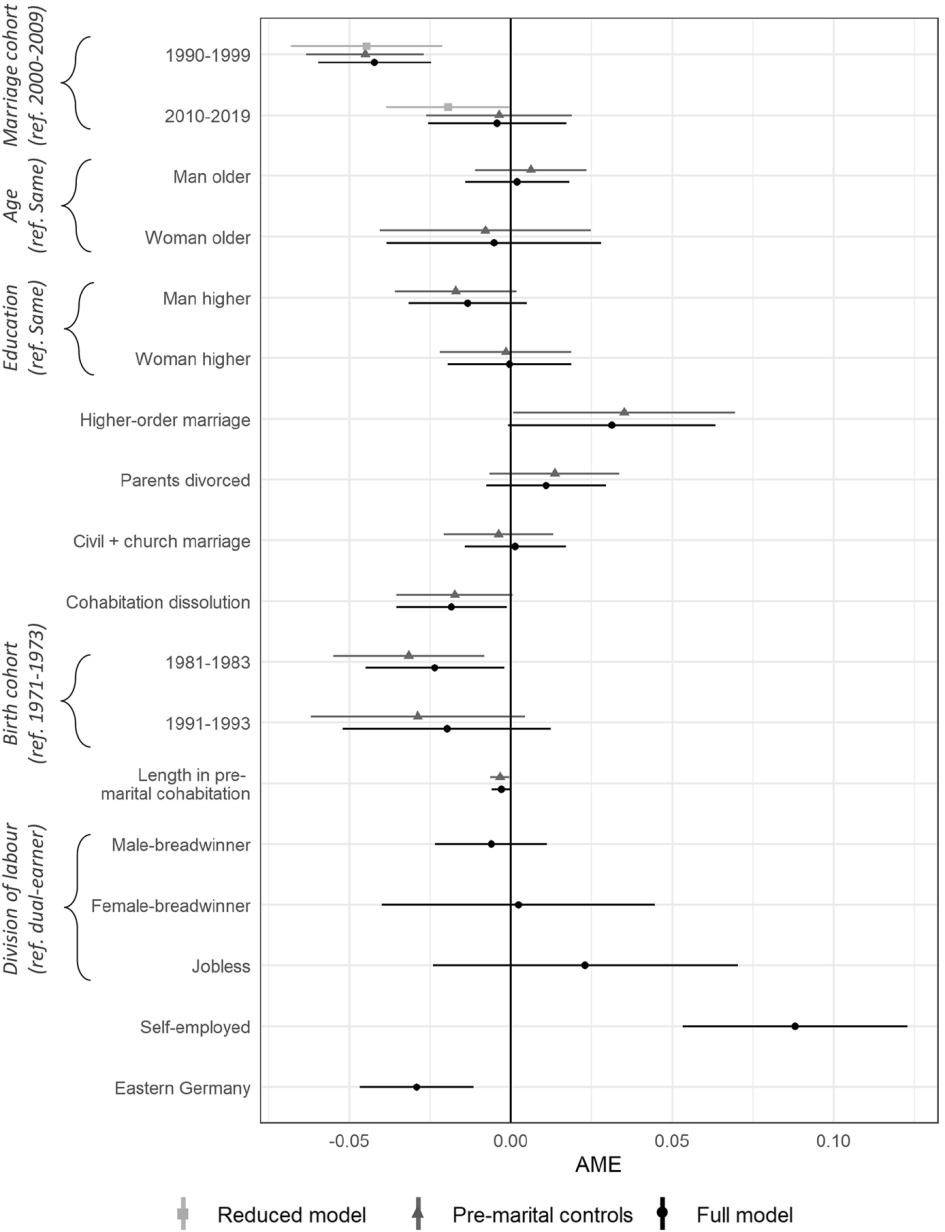
Complementary log–log models predicting the prevalence of a marital contract (including all contracts) *Note*: Lines indicate 95% confidence intervals. AME: Average Marginal Effects

Next, we turn to a complementary log–log model to predict the probability of a marital contract. Figure 2 presents average marginal effects (AMEs). We differentiate between the reduced model, which only includes marriage cohorts as predictors, a model with pre-marital predictors, and the full model, including both pre- and post-marital predictors (also see Table A3 in the online appendix for coefficient estimates). The AMEs of the marriage cohorts show a statistically significant negative association with marital contracts, indicating that when other factors are not controlled, couples married in the 1990s and 2010s are significantly less likely to have a marital contract compared with the 2000s marriage cohort. Those married in the 1990s are 4.5 percentage points and those married in the 2010s are 2.0 points less likely to have marital contracts than those married in the 2000s (reference group). Controlling for the additional predictors, couples married in the 2010s are equally likely to have a marital contract than those married in the 2000s. For couples married in the 1990s, this probability remains significantly lower at 4.5 percentage points in the model including pre-marital characteristics and 4.2 points in the full model.

The sequential inclusion of the coefficients in the model shows that the association between marriage cohorts and marital contracts is partly driven by differences in the age composition of couples with and without contracts and partly by a lack of postnuptial contracts that have not yet been signed in more recent marriage cohorts (see Figure A2 in the online appendix). Postnuptial contracts are signed throughout marriage, for instance when the employment or family situations of spouses change. As this might not have been the case for younger marriage cohorts, we may underestimate the proportion of marital contracts in these cohorts. Further, younger marriage cohorts also tend to be younger, on average, thus being less likely to have experienced union dissolution, which might additionally increase the prevalence among these cohorts in the future.

Against our expectations, we do not find within-couple differences in age, education, or employment to be clearly associated with marital contracts. Self-employment is the strongest statistically significant predictor of couples having signed a marital contract. In couples with at least one self-employed spouse, marital contracts are 8.8 percentage points more likely than in couples not in self-employment. Further, marital contracts are more likely at 3.1 percentage points in higher-order marriages compared with couples in their first marriage (significant at the 10% level). Against our expectations, the experience of a cohabitation dissolution is associated with a statistically significant decrease of 1.8 percentage points of the probability of having signed a marital contract. We can only speculate about potential explanations, but those who went through trial marriages before may be more certain about their current partner choice. Significantly different from couples in Western Germany, couples in Eastern Germany are 2.9 percentage points less likely to make a marital contract. This indicates that individualistic couples in the east are more likely to remain in unmarried cohabitation, whereas these couples might be more likely to enter a modified marriage in the west. Both parental divorce and the type of marriage ceremony are not significantly associated with having a contract.

**Fig. 3:**
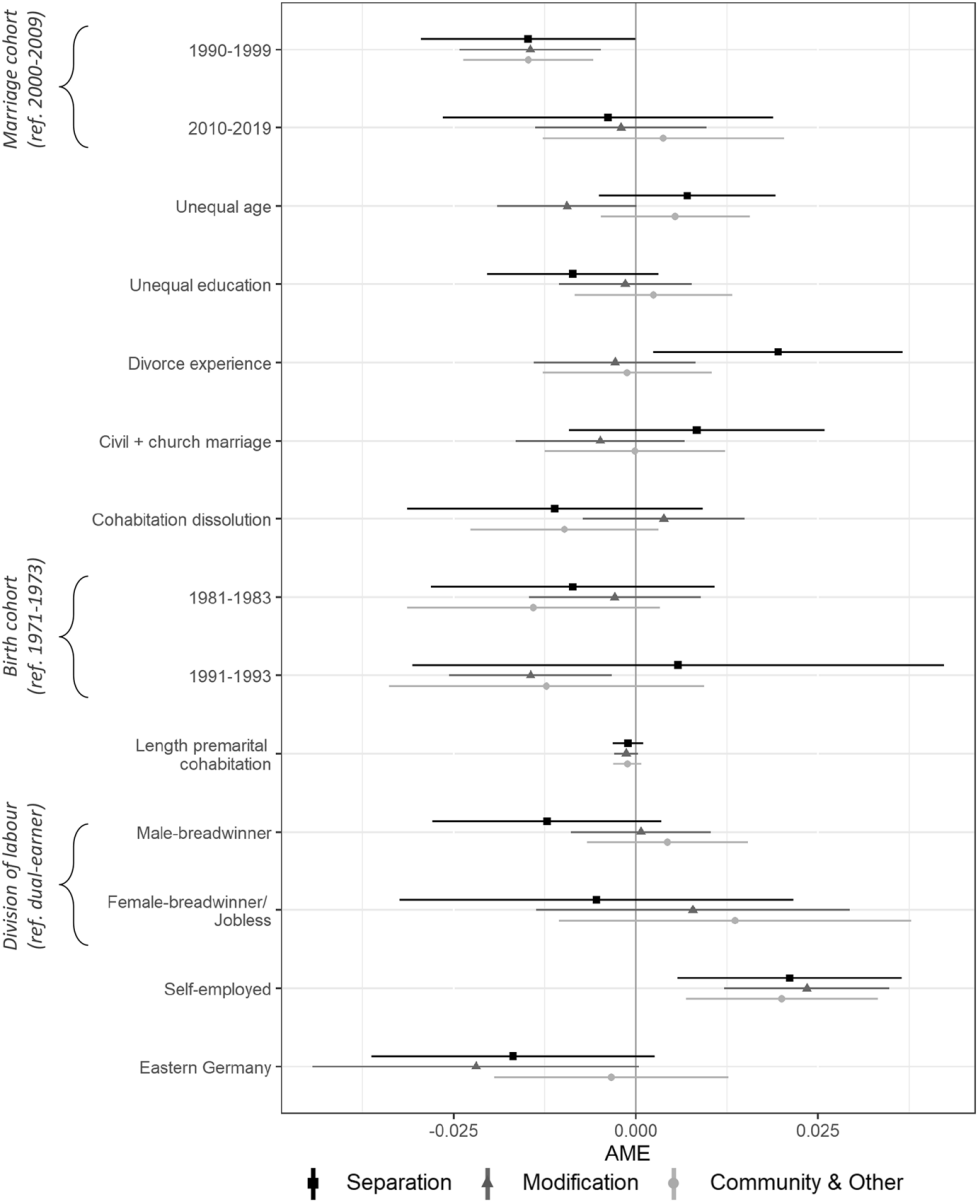
Multinomial logistic regression model predicting the prevalence of type of marital contract (reference group: no contract) *Note*: Lines indicate 95% confidence intervals. AME: Average Marginal Effects; Separation: separation of property; Modification: modified community of accrued gain; Community & Other: community of property or other contract type

In the following, we examine the predictors of the type of marital contract, differentiating between the separation of property, a modified community of accrued gains, the community of property together with other contracts, and no contract (reference group). Figure 3 shows average marginal effects (AMEs) of a multinomial logistic regression model (also see Table A4 in the online appendix for coefficient estimates). Due to sparse cell sizes, we summarise variables on age and educational differences (same (reference group) or unequal), own and parental divorce experience, and the division of labour (dual-earner (reference group), male-breadwinner, and female-breadwinner/jobless) in the following regression model.

We find that couples married in the 1990s are significantly less likely to have marital contracts of any type than those married in the 2000s (reference group), indicating a generally lower prevalence of marital contracts in older marriage cohorts. No significant differences exist between couples married in the 2000s and the 2010s. Within-couple differences in age, education, and the division of labour are not significantly associated with marital contracts—except for agreements on the community of property or other contracts being 0.9 percentage points less likely than having no contract in couples with unequal age compared with couples of same age (reference group). As this association is only significant at the 10% level, the results overall suggest that resource differentials between spouses are not clearly associated with the type of marital contract.

In return, the experience of the own or parental divorce is significantly positively associated with the separation of property, suggesting that these couples favour individualised marriages. Further, couples with at least one self-employed partner are significantly more likely to opt for a marital contract of any type than couples without a self-employed spouse. Having marital contracts of different types might reflect different needs and preferences of self-employed spouses to regulate the consequences of self-employment within marriage—potentially ranging from the separation of business assets and other property to full asset pooling if both spouses own the business jointly.

## Discussion

In this study, we highlight the relevance of matrimonial property regimes in modern societies that define the legal status of marriage and have far-reaching economic consequences for women and men both during marriage and in the event of divorce (Dutta, 2012). However, the prevalence and the predictors of marital contracts to opt out of the default regime remain unclear in many countries, including Germany (Rainer, 2007). We test expectations about cohort changes and within-couple predictors of marital contracts in Germany, a country with the community of accrued gains as a unique default matrimonial property regime. Drawing on rich survey data from an interview module implemented in pairfam (2018/19), we employ complementary log–log and multinominal logistic regression models to predict the prevalence and the type of marital contracts.

We show that 5% of all married couples opt out of the default regime in Germany. This finding indicates that a ‘customisation’ of the economic outcomes of divorce is broadly uncommon among German couples. Differentiating between contract types, most couples either specify a separation of property (40%) or modify the community of accrued gains (31%). In line with the widespread notion of marital contracts as expression of individuality and independence (Thompson, 2018), only around 18% of all contracts agree on a community of property.

Building on literature on the individualisation of marriage (Cherlin, 2004), we find an increase in marital contracts across marriage cohorts, with cohorts from the 2000s and 2010s being more likely to have signed a contract than earlier cohorts. With proportions below 10%, however, marital contracts are still rare in Germany compared with other countries. The low proportion might reflect the German default matrimonial property regime that has existed since 1957 without major reforms, whereas other European countries, such as France or Spain, have experienced a customisation of marriage during the last decades (Brassiolo, 2013; Frémeaux & Leturcq, 2018).

We present the finding that being in a higher-order marriage is positively associated with opting out of the default. Individuals who experienced a divorce are more likely to choose the separation of property compared with those without divorce experience. Further, in line with prior research on the Italian case (Fraboni & Vitali, 2019; Ruiu & Breschi, 2017), we find that self-employment of at least one partner is positively associated with opting out of the default. Our results also show that couples in Eastern Germany are less likely to make a marital contract than those in Western Germany. Against our expectations, age differences between spouses as well as the division of labour are not clearly associated with the choice to opt out of the default regime.

Germany’s low share of couples signing a marital contract illustrates the importance of considering the interplay of the institutional context and the matrimonial property regime in future research on marriage and divorce. In contrast to findings from Fraboni and Vitali (2019) for Italy, most couples in Germany do not consider opting out of the default irrespective of their economic resources and the division of labour. Although the prevalence of marital contracts has increased slightly, high costs of a notarially certified marital contract, little knowledge about the default matrimonial property regime, and consequences of modifications might explain the low prevalence of marital contracts in Germany (Wippermann, 2013).

Our results suggest that two distinct types of incentives lead couples to opt for a separation of property. First, self-employment underlines the importance of circumventing economic conflicts during marriage and protecting both spouses’ property from the other’s legal compensation claims both during and after marriage. Thus, marital contracts among the self-employed might be understood less as expression of individualisation but as customisation to secure business assets and to exclude the non-self-employed spouse, most often the woman, from any liability obligations. Second, prior divorce experiences may raise awareness of the potential consequences of divorce. With this knowledge, individuals might be better able to assess the possible benefits of a customisation of their marriage with a contract. Our results show that divorce experiences are positively associated with the separation of property. The results illustrate the relevance of marital contracts as a further potential mechanism of the intergenerational transmission of divorce because marital contracts can reduce the costs of divorce (Amato et al., 2007).

Drawing on rich survey data, we generate important insights into the prevalence and the predictors of marital contracts in Germany, but some limitations of our study need to be acknowledged. First, due to the cross-sectional nature of our data, we were not able to consider spouses’ resource differentials prior to signing their marital contracts. The results on the predictors of marital contracts based on our study should therefore be exclusively understood as descriptive. To make the link between couple characteristics and signing a marital contract clearer, future research should consider resource differentials at the time of contract conclusion. Second, pairfam data do not include individual-level measures of economic resources such as housing or business assets. As proxy variables for resource differentials within couples, we therefore included differences in age and education between partners instead of more volatile income measures. Third, the small sample size might reduce the accuracy of our estimates.

Our findings are relevant considering the far-reaching social policy implications of marital contracting. Welfare expenditures following divorce are mostly caused by transfers to people in need (Schramm et al., 2013). The institution of marriage potentially ensures the economic protection of the weaker spouse by applying the principle of ‘spouse subsidiarity’. In case of one spouse’s indigence, most welfare transfers are only paid if the other spouse does not have the financial means to provide support. If individuals, most often women, cannot afford the necessities to sustain the household after divorce, the welfare state steps in to provide social security benefits. For the USA, the annual economic burden amounts to $300 million caused by welfare state expenditures due to divorce (Schramm, 2006).

Marital contracts can be seen as an effective instrument in governing the economic outcomes of divorce for both individuals and the state. This is especially true for Germany, where the default community of accrued gains strongly disadvantages the spouse, most often the wife, focusing on household labour. The primary earner, most often the husband, is entitled to dispose of most assets freely because the distribution of surplus money takes place only in the event of divorce. This unequal say could cause severe disadvantages in asset allocation during marriage and thus lead to a lack of the wife’s financial means after divorce. Particularly in the prevalent male-breadwinner/female-carer couples in Germany, wives are disadvantaged as they cannot intercede in case of the husband’s wealth mismanagement. In addition, their bargaining power in marriage might be restricted due to the limited access to the husband’s wealth. Most wives remain restricted in their ability to access and accumulate economic resources, which might prevent them from leaving an unhappy marriage due to the financial restrictions they face. Family law scholars therefore demand to reform the German default regime following the community of acquisitions, which is in place in many European countries and allows the legal participation in the spouse’s wealth already during marriage (Nake, 2013). This reform is especially relevant in light of the pronounced gender gap in individual wealth in Germany that is particularly strong in couples with gender-traditional employment arrangements (Grabka et al., 2015; Nutz & Gritti, 2021). Additional policies targeting misconceptions about matrimonial property regimes by providing information could further reduce these gender wealth inequalities during and after marriage.

## Supplementary Information

Below is the link to the electronic supplementary material.

Supplementary file1 (PDF 384 kb)

## Footnotes

1 Other sources of survey data include the Socio-Economic Panel Innovation Study 2016 (Richter and Schupp 2015), the Socio-Economic Panel Core Study 1988 and 2019 (doi:10.5684/soep.core.v36eu) and data from the Federal Ministry of Family Affairs, Senior Citizens, Women and Youth from 2010 (Federal Ministry for Family Affairs, Senior Citizens, Women and Youth of Germany 2014).

2 Other European countries with a community of accrued gains are Cyprus and Greece (Rotino 2015).

3 Surplus gains are calculated as half of the difference between the gains of both spouses accrued throughout marriage (Dutta 2012).

4 A detailed description of the pairfam study can be found in Huinink et al. (2011).

5 Pairfam data do not include information on parental divorce and cohabitation dissolution from the partner.

6 Binary logistic regression models provide similar results (see Table A1 in the online appendix).

## References

[CR1] Amato, P. R., Booth, A., Johnson, D. R., & Rogers, S. J. (2007). *Alone together: How marriage in America is changing*. Harvard University Press.

[CR2] Bayot, D., & Voena, A. (2014). *Prenuptial contracts, labor supply and household investments*. University of Chicago.

[CR3] Becker GS (1973). A theory of marriage: Part I. Journal of Political Economy.

[CR4] Billari FC, Liefbroer AC (2016). Why still marry? The role of feelings in the persistence of marriage as an institution. The British Journal of Sociology.

[CR5] Blood, R. O., & Wolfe, D. M. (1960). *Husbands and wives: The dynamics of married living*. The Free Press.

[CR6] Brassiolo, P. (2013). *The effect of property division laws on divorce and labor supply: Evidence from Spain* (CAF Working Papers 2013/02).

[CR7] Brüderl, J., Drobnič, S., Hank, K., Neyer, F. J., Walper, S., Alt, P., et al. (2020). *The German Family Panel (pairfam)*. GESIS Data Archive, Cologne. ZA5678 Data file Version 11.0.0.

[CR8] Burgoyne C, Morison V (1997). Money in remarriage: Keeping things simple—and separate. The Sociological Review.

[CR9] Cherlin AJ (2004). The deinstitutionalization of American marriage. Journal of Marriage and Family.

[CR11] Deere CD, Doss CR (2006). The gender asset gap: What do we know and why does it matter?. Feminist Economics.

[CR12] Dingeldey I (2001). European tax systems and their impact on family employment patterns. Journal of Social Policy.

[CR13] Dutta, A. (2012). Marital agreements and private autonomy in Germany. In J. M. Scherpe (Ed.), *Marital agreements and private autonomy in comparative perspective* (pp. 158–199). Hart Publishing.

[CR14] Eurostat (2021). *Crude marriage rate and crude divorce rate.* Retrieved December 27, 2021, from https://ec.europa.eu/eurostat/web/products-datasets/product?code=tps00206

[CR10] Federal Ministry for Family Affairs, Senior Citizens, Women and Youth of Germany (2014). *Partnerschaft und Ehe - Entscheidungen im Lebensverlauf: Einstellungen, Motive, Kenntnisse des rechtlichen Rahmens [Partnership and marriage – Decisions in the course of life: Attitudes, motives, knowledge of the legal framework]*.

[CR16] Fraboni R, Vitali A (2019). Gender differences in couples' matrimonial property regime in Italy. Journal of Marriage and Family.

[CR17] Frémeaux N, Leturcq M (2018). Prenuptial agreements and matrimonial property regimes in France, 1855–2010. Explorations in Economic History.

[CR18] Goebel J, Grabka MM, Liebig S, Kroh M, Richter D, Schröder C (2019). The German socio-economic panel (SOEP). Journal of Economics and Statistics.

[CR19] Grabka MM, Marcus J, Sierminska E (2015). Wealth distribution within couples. Review of Economics of the Household.

[CR20] Hamilton V (2004). Mistaking marriage for social policy. Virginia Journal of Social Policy and the Law.

[CR21] Honaker J, King G, Blackwell M (2011). Amelia II: A program for missing data. Journal of Statistical Software.

[CR22] Huinink J, Brüderl J, Nauck B, Walper S, Castiglioni L, Feldhaus M (2011). Panel analysis of intimate relationships and family dynamics (pairfam): Conceptual framework and design. Journal of Family Research.

[CR23] King G, Zeng L (2001). Logistic regression in rare events data. Political Analysis.

[CR24] Langenfeld, G., & Milzer, L. (2019). *Handbuch der Eheverträge und Scheidungsvereinbarungen [Handbook of prenuptial agreements and divorce settlements]* (8th ed.). Beck.

[CR25] Mahar, H. (2003). *Why are there so few prenuptial agreements?* Harvard Law School John M. Olin Center for Law, Economicsand Business Discussion Paper Series Paper 436. http://www.law.harvard.edu/programs/olin_center/papers/pdf/436.pdf. Accessed 5 January 2021.

[CR26] Marston AA (1997). Planning for love: The politics of prenuptial agreements. Stanford Law Review.

[CR27] Nake, A. (2013). Statement aus Sicht des Deutschen Juristinnenbundes. In G. Brudermüller, B. Dauner-Lieb, & S. Meder (Eds.), *Wer hat Angst vor der Errungenschaftsgemeinschaft? Auf dem Weg zu einem partnerschaftlichen Güterrecht – Schlussfolgerungen aus dem 1. Gleichstellungsbericht [Who is afraid of the community of acquisitions? On the way to a partnership-based property law – Conclusions from the First Equality Report]* (1st ed., pp. 93–98). V&R Unipress.

[CR28] Nutz T, Gritti D (2021). Dyadic employment biographies and within-couple wealth inequality in Britain and Western Germany. Journal of Marriage and Family.

[CR29] Page, A. (2001). *Marriage in the European Union today*. Briefing 2. National Family & Parenting Institute.

[CR30] R Core Team (2020). *R: A language and environment for statistical computing*.

[CR31] Radenacker A, Kreyenfeld M, Stracke E, Mika T (2019). Der Ausschluss des Versorgungsausgleichs: Hintergründe und Trends [The exclusion of pension equalisation: Background and trends]. Neue Zeitschrift für Familienrecht.

[CR32] Rainer H (2007). Should we write prenuptial contracts?. European Economic Review.

[CR33] Richter D, Schupp J (2015). The SOEP Innovation Sample (SOEP IS). Schmollers Jahrbuch.

[CR34] Rotino, S. (2015). *Der gesetzliche Güterstand im europäischen Vergleich: Arbeitspapier für die Sachverständigenkommission zum Zweiten Gleichstellungsbericht der Bundesregierung*. *[The statutory matrimonial property regime in a European comparison: Working paper for the Expert Commission on the Federal Government’s Second Report on Equality.]*

[CR35] Rowlingson, K., & Joseph, R. (2010). *Assets and debts within couples: Ownership and decision-making*. Friends Provident Foundation.

[CR36] Rubin DB (1976). Inference and missing data. Biometrika.

[CR37] Ruiu G, Breschi M (2017). “Let’s talk about love”: An analysis of the religious and economic factors determining the choice of marital property regime in Italy. Demographic Research.

[CR38] Schramm DG (2006). Individual and social costs of divorce in Utah. Journal of Family and Economic Issues.

[CR39] Schramm DG, Harris SM, Whiting JB, Hawkins AJ, Brown M, Porter R (2013). Economic costs and policy implications associated with divorce: Texas as a case study. Journal of Divorce and Remarriage.

[CR40] Schreiber, M. (1983). *Gestaltungsfreiheit in Eheverträgen in rechtsdogmatischer und rechtstatsächlicher Sicht [Freedom of organisation in marriage contracts from a legal doctrinal and factual point of view]*. PhD thesis. University of Constance.

[CR41] Smith I (2003). The law and economics of marriage contracts. Journal of Economic Surveys.

[CR42] Stach, S. (1988). *Eheverträge - Gesetz und Rechtstatsachen [Prenuptial agreements – Law and legal facts]*. PhD thesis. Free University of Berlin.

[CR43] Thompson S (2018). Feminist relational contract theory: A new model for family property agreements. Journal of Law and Society.

[CR44] Van de Kaa D (1987). Europe’s second demographic transition. Population Bulletin.

[CR45] Vogler C, Brockmann M, Wiggins RD (2006). Intimate relationships and changing patterns of money management at the beginning of the twenty-first century. The British Journal of Sociology.

[CR46] Wippermann, C. (2013). Partnerschaft und Ehe im Lebensverlauf – Die Rechtsfolgen von Heirat und Scheidung in der empirischen Sozialforschung [Partnership and marriage over the life course – The legal consequences of marriage and divorce in empirical social research]. In G. Brudermüller, B. Dauner-Lieb, & S. Meder (Eds.), *Wer hat Angst vor der Errungenschaftsgemeinschaft?*: *Auf dem Weg zu einem partnerschaftlichen Güterrecht – Schlussfolgerungen aus dem 1. Gleichstellungsbericht Gleichstellungsbericht [Who is afraid of the community of acquisitions? On the way to a partnership-based property law – Conclusions from the First Equality Report]*, 1st ed., (pp. 23–40). V&R Unipress.

[CR47] Yodanis C, Lauer S (2014). Is marriage individualized? What couples actually do. Journal of Family Theory and Review.

