# Supplementary material for: Who Opts Out? The Customisation of Marriage in the German Matrimonial Property Regime
Source: Eur J Popul. 2022 Mar 23;38(3):353–75. doi: 10.1007/s10680-022-09613-8 (PMC9363548; doi:10.1007/s10680-022-09613-8)
Supplement: Supplementary file 1 — Supplementary file1 (PDF 384 kb) [file 10680_2022_9613_MOESM1_ESM.pdf]

Who Opts Out? The Customisation of Marriage in the German Matrimonial Property Regime

Online Appendix

Table A1. Logistic regression model on all marital contracts

	(1)
Marriage cohort (ref. 2000-2009)	
1990-1999	-1.517 ***
	(0.453)
2010-2019	-0.102
	(0.226)
Age difference (ref. same age)	
Man older	0.042
	(0.192)
Woman older	-0.128
	(0.438)
Educational difference (ref. same)	
Man higher	-0.355
	(0.259)
Woman higher	-0.024
	(0.218)
Higher-order marriage (ref. first marriage)	0.627 *
	(0.267)
Parents divorced (ref. not divorced)	0.246
	(0.246)
Civil and church marriage (ref. civil)	0.036
	(0.225)
Cohabitation dissolution (ref. no dissolution)	-0.473 *
	(0.241)
Birth cohort (ref. 1971-1973)	
1981-1983	-0.507 *
	(0.225)
1991-1993	-0.394
	(0.366)
Length in premarital cohabitation	-0.070 *
	(0.034)
Division of labour (ref. dual-earner)	
Male-breadwinner	-0.135
	(0.205)
Female-breadwinner	0.065
	(0.462)
Jobless	0.427
	(0.391)
Self-employed (ref. not self-employed)	1.406 ***
	(0.200)
Eastern Germany (ref. Western Germany)	-0.823 *
	(0.336)
Intercept	-2.473 ***
	(0.308)
N	2880

Note: Coefficient estimates of logistic regression models for all contracts. Standard errors in parentheses. *** p<0.001; ** p<0.01; * p<0.05.
Source: pairfam (wave 11; weighted by post-stratification weights, multiply imputed).

Table A2. Overview of variables used for regression analyses including number and share of missing values

	Missing values	
	Number	%
Type of marriage	1,072	37.22
Parental divorce	1,065	36.98
Higher-order marriage	79	2.74
Cohabitation dissolution	76	2.64
Age difference	31	1.08
Length in premarital cohabitation	23	0.80
Education difference	14	0.49
Division of labour	11	0.38
Self-employed	9	0.31
Marriage cohort	2	0.07
Eastern Germany	1	0.00
Birth cohort	0	0.00

Figure A1. Prevalence of marital contracts among divorced and married subpopulation

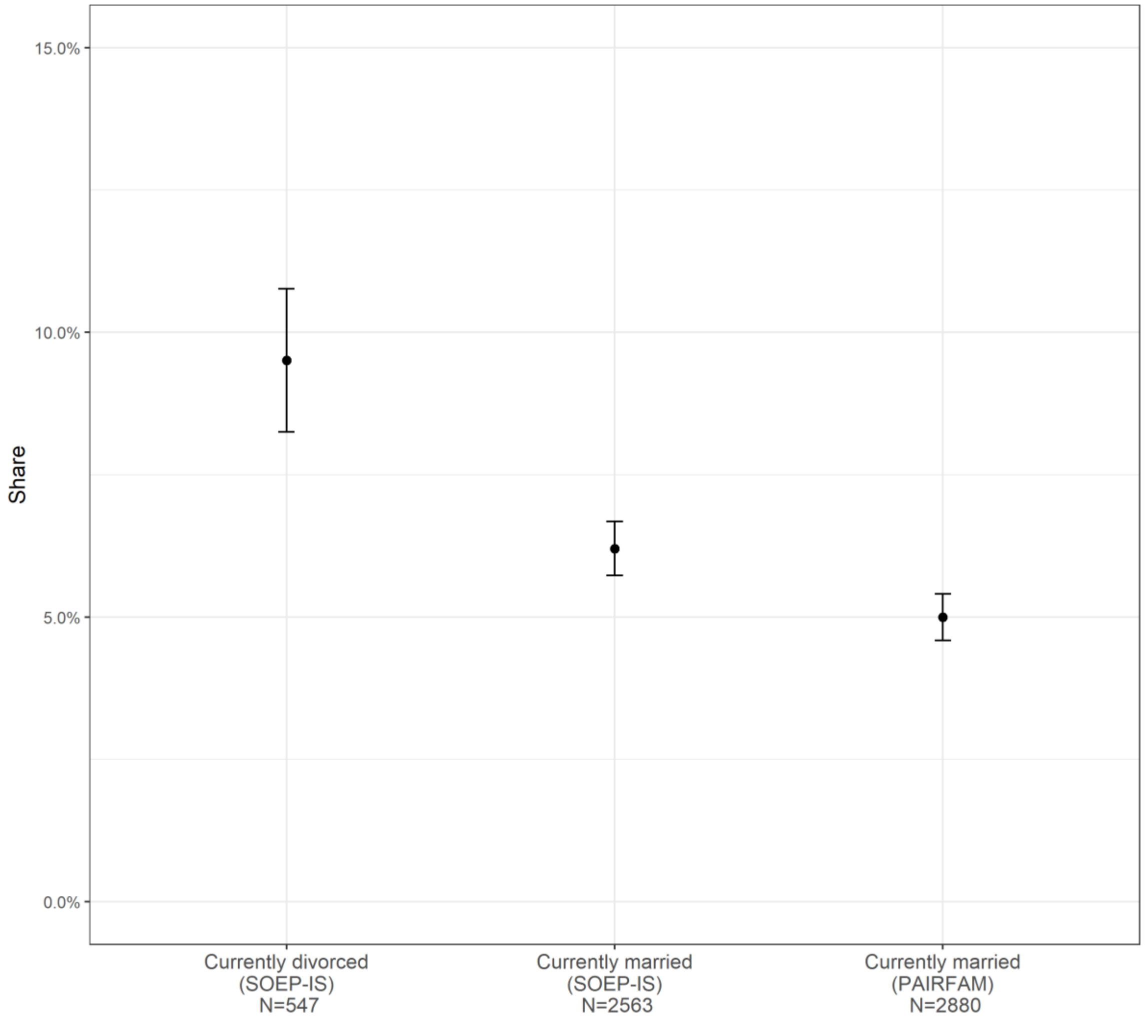

Note: Bars indicate 95% confidence intervals.

Figure A2. Prevalence of pre- and postnuptial marital contracts across marriage cohorts

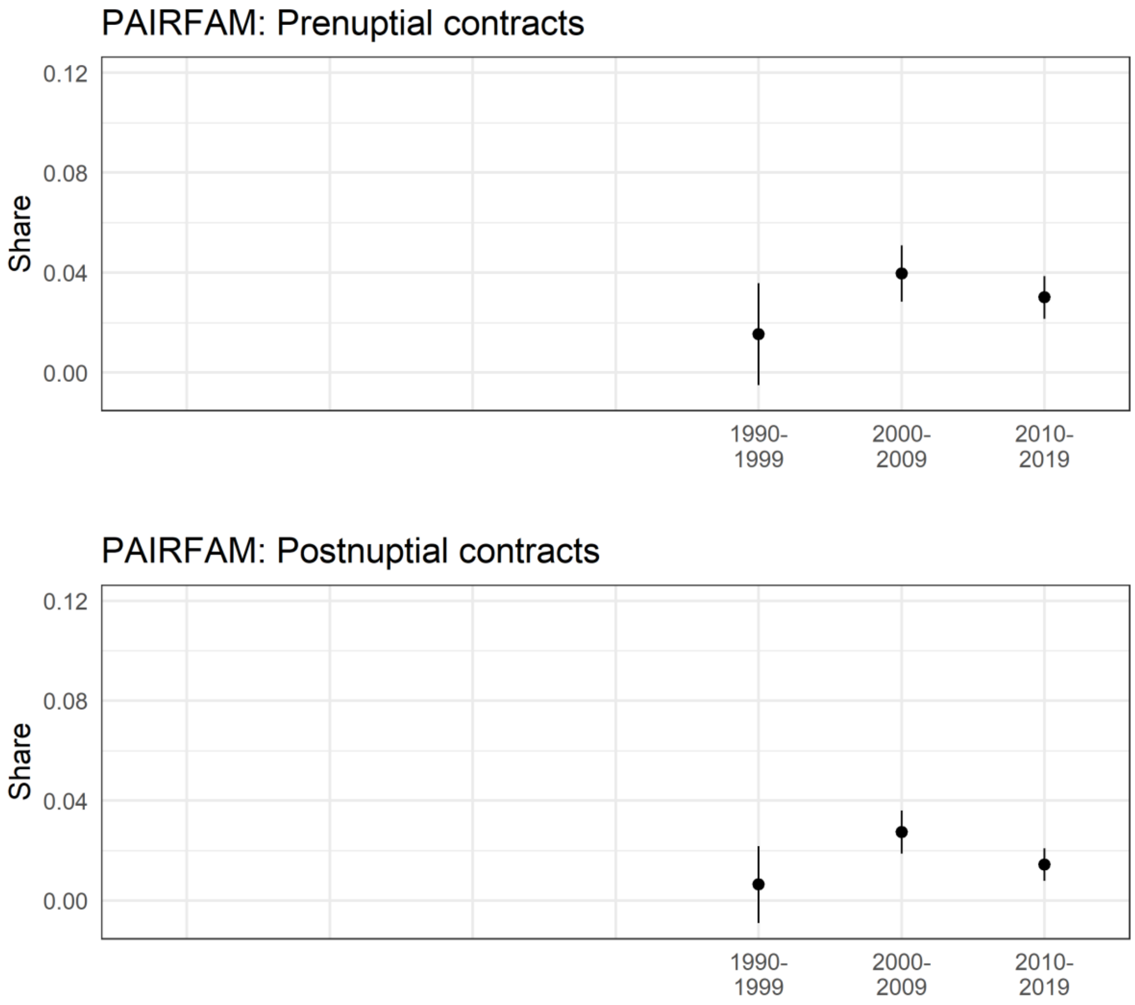

Note: Bars indicate 95% confidence intervals.

Table A3. Complementary log-log models on all marital contracts

	Coefficients			Average Marginal Effects		
	(1)	(2)	(3)	(1)	(2)	(3)
Marriage cohort (ref. 2000-2009)						
1990-1999	-1.145**	-1.487***	-1.459***	-0.045***	-0.045***	-0.042***
	(0.428)	(0.440)	(0.442)	(0.012)	(0.009)	(0.009)
2010-2019	-0.358*	-0.066	-0.082	-0.019*	-0.004	-0.004
	(0.173)	(0.211)	(0.216)	(0.010)	(0.012)	(0.011)
Age difference (ref. same)						
Man older		0.126	0.044		0.006	0.002
		(0.180)	(0.182)		(0.009)	(0.008)
Woman older		-0.186	-0.124		-0.008	-0.005
		(0.425)	(0.424)		(0.017)	(0.017)
Educational difference (ref. same)						
Man higher		-0.398	-0.324		-0.017†	-0.013
		(0.248)	(0.248)		(0.010)	(0.009)
Woman higher		-0.031	-0.010		-0.002	0.000
		(0.207)	(0.207)		(0.010)	(0.010)
Higher-order marriage (ref. first marriage)		0.597*	0.574*		0.035*	0.031†
		(0.246)	(0.251)		(0.018)	(0.016)
Parents divorced (ref. not divorced)		0.261	0.228		0.014	0.011
		(0.234)	(0.233)		(0.010)	(0.009)
Civil and church marriage (ref. civil)		-0.078	0.032		-0.004	0.001
		(0.214)	(0.216)		(0.009)	(0.008)
Cohabitation dissolution (ref. no dissolution)		-0.392	-0.446		-0.017†	-0.018*
		(0.224)	(0.230)		(0.009)	(0.009)
Birth cohort (ref. 1971-1973)						
1981-1983		-0.603**	-0.489*		-0.032**	-0.024*
		(0.210)	(0.215)		(0.012)	(0.011)
1991-1993		-0.533	-0.396		-0.029†	-0.020
		(0.341)	(0.352)		(0.017)	(0.016)

Table A3, continued						
	Coefficients			Average Marginal Effects		
	(1)	(2)	(3)	(1)	(2)	(3)
Length in premarital cohabitation		-0.070*	-0.066*		-0.003*	-0.003*
		(0.032)	(0.032)		(0.002)	(0.001)
Division of labour (ref. dual-earner)						
Male-breadwinner			-0.136			-0.006
			(0.194)			(0.009)
Female-breadwinner			0.049			0.002
			(0.438)			(0.022)
Jobless			0.404			0.023
			(0.376)			(0.024)
Self-employed (ref. not self-employed)			1.323***			0.088***
			(0.189)			(0.018)
Eastern Germany (ref. Western Germany)			-0.801*			-0.029**
			(0.329)			(0.009)
Intercept	-2.681***	-2.259***	-2.524***			
	(0.128)	(0.234)	(0.293)			
N		2880			2880	

Note: Presented are coefficient estimates and average marginal effects of complementary log-log models for all contracts. Standard errors in parentheses. *** p<0.001; ** p<0.01; * p< 0.05; †p< 0.1.
Source: pairfam (wave 11; weighted by post-stratification weights, multiply imputed).

Table A4. Multinomial logistic regression models by types of marital contracts (reference: no contract)

	Coefficients			Average Marginal Effects		
	Separation	Modification	Community / Other	Separation	Modification	Community / Other
Marriage cohort (ref. 2000-2009)						
1990-1999	-0.987†	-2.364*	-1.877†	-0.015*	-0.015**	-0.015**
	(-1.80)	(-2.19)	(-1.80)	(-1.97)	(-3.25)	(-2.92)
2010-2019	-0.172	0.205	-0.130	-0.004	0.004	-0.002
	(-0.33)	(0.44)	(-0.34)	(-0.33)	(0.45)	(-0.34)
Age difference (ref. same)						
Unequal age	0.344	0.355	-0.700†	0.007	0.005	-0.009†
	(1.12)	(1.03)	(-1.80)	(1.14)	(1.05)	(-1.93)
Educational difference (ref. same)						
Unequal education	-0.453	0.144	-0.115	-0.009	0.002	-0.001
	(-1.42)	(0.39)	(-0.33)	(-1.44)	(0.44)	(-0.31)
Divorce experience (ref. no divorce experience)	0.832**	-0.0529	-0.188	0.020*	-0.001	-0.003
	(2.73)	(-0.13)	(-0.41)	(2.24)	(-0.20)	(-0.51)
Civil and church marriage (ref. civil)	0.413	-0.004	-0.340	0.008	-0.000	-0.005
	(0.95)	(-0.01)	(-0.80)	(0.94)	(-0.02)	(-0.83)
Cohabitation dissolution (ref. no dissolution)	-0.569	-0.670	0.242	-0.011	-0.010	0.004
	(-1.10)	(-1.58)	(0.59)	(-1.08)	(-1.49)	(0.68)
Birth cohort (ref. 1971-1973)						
1981-1983	-0.480	-0.861*	-0.228	-0.009	-0.014	-0.003
	(-0.97)	(-2.01)	(-0.60)	(-0.87)	(-1.59)	(-0.48)
1991-1993	0.182	-0.708	-1.761*	0.006	-0.012	-0.015*
	(0.26)	(-1.11)	(-2.12)	(0.31)	(-1.11)	(-2.54)
Length in premarital cohabitation	-0.058	-0.082	-0.101†	-0.001	-0.000	-0.001
	(-1.08)	(-1.29)	(-1.69)	(-0.98)	(-1.18)	(-1.57)
Division of labour (ref. dual-earner)						
Male-breadwinner	-0.571	0.307	0.044	-0.012	0.004	0.001
	(-1.63)	(0.69)	(0.12)	(-1.52)	(0.78)	(0.15)
Female-breadwinner/Jobless	-0.185	0.809	0.503	-0.005	0.014	0.008
	(-0.31)	(1.34)	(0.83)	(-0.39)	(1.10)	(0.71)
Self-employed (ref. not self-employed)	1.135**	1.428***	1.780***	0.021**	0.020**	0.024***
	(2.99)	(3.86)	(4.83)	(2.69)	(2.98)	(4.06)
Eastern Germany (ref. Western Germany)	-0.890†	-0.296	-1.629*	-0.017†	-0.003	-0.022†
	(-1.91)	(-0.54)	(-2.11)	(-1.70)	(-0.41)	(-1.92)

Table A4, continued

	Coefficients			
	Separation	Modification	Community / Other	
Intercept	-3.577**	-4.476***	-3.522**	
	(-2.58)	(-3.77)	(-2.74)	
N		2880		2880

Note: Presented are coefficient estimates and average marginal effects. Reference category: no contract. Standard errors in parentheses. *** p<0.001; ** p<0.01; * p<0.05; †p< 0.1.
Source: pairfam (wave 11; weighted by post-stratification weights, multiply imputed).
